# SARS-CoV-2 infection and pulmonary tuberculosis in children and adolescents: a case-control study

**DOI:** 10.1186/s12879-023-08412-8

**Published:** 2023-06-29

**Authors:** Jeremi Swanepoel, Marieke M. van der Zalm, Wolfgang Preiser, Gert van Zyl, Elizabeth Whittaker, Anneke C. Hesseling, David A. J. Moore, James A. Seddon

**Affiliations:** 1 Desmond Tutu TB Centre, Department of Paediatrics and Child Health, Faculty of Medicine and Health Sciences, Stellenbosch University, Stellenbosch, South Africa; 2 TB Centre, London School of Hygiene and Tropical Medicine, London, UK; 3 Division of Medical Virology, Department of Pathology, Faculty of Medicine and Health Sciences, Stellenbosch University and National Health Laboratory Service, Tygerberg Academic Hospital, Cape Town, South Africa; 4 Department of Infectious Disease, Imperial College London, London, UK

**Keywords:** Tuberculosis, SARS-CoV-2, Adolescents, Immunology

## Abstract

**Background:**

The Severe Acute Respiratory Syndrome-Coronavirus-2 (SARS-CoV-2) pandemic has had an impact on the global tuberculosis (TB) epidemic but evidence on the possible interaction between SARS-CoV-2 and TB, especially in children and adolescents, remains limited. We aimed to evaluate the relationship between previous infection with SARS-CoV-2 and the risk of TB in children and adolescents.

**Methods:**

An unmatched case-control study was conducted using SARS-CoV-2 unvaccinated children and adolescents recruited into two observational TB studies (Teen TB and Umoya), between November 2020 and November 2021, in Cape Town, South Africa. Sixty-four individuals with pulmonary TB (aged < 20 years) and 99 individuals without pulmonary TB (aged < 20 years) were included. Demographics and clinical data were obtained. Serum samples collected at enrolment underwent quantitative SARS-CoV-2 anti-spike immunoglobulin G (IgG) testing using the Abbott SARS-CoV-2 IgG II Quant assay. Odds ratios (ORs) for TB were estimated using unconditional logistic regression.

**Results:**

There was no statistically significant difference in the odds of having pulmonary TB between those who were SARS-CoV-2 IgG seropositive and those who were seronegative (adjusted OR 0.51; 95% CI: 0.23–1.11; n = 163; p = 0.09). Of those with positive SARS-CoV-2 serology indicating prior infection, baseline IgG titres were higher in individuals with TB compared to those without TB (p = 0.04) and individuals with IgG titres in the highest tertile were more likely to have pulmonary TB compared to those with IgG levels in the lowest tertile (OR: 4.00; 95%CI: 1.13– 14.21; p = 0.03).

**Conclusions:**

Our study did not find convincing evidence that SARS-CoV-2 seropositivity was associated with subsequent pulmonary TB disease; however, the association between magnitude of SARS-CoV-2 IgG response and pulmonary TB warrants further investigation. Future prospective studies, evaluating the effects of sex, age and puberty on host immune responses to *M. tuberculosis* and SARS-CoV-2, will also provide more clarity on the interplay between these two infections.

**Supplementary Information:**

The online version contains supplementary material available at 10.1186/s12879-023-08412-8.

## Background

According to the recent World Health Organization (WHO) Global TB report, over one million children younger than 15 years of age fell ill with tuberculosis (TB) in 2021 [1]. It is estimated that a quarter of these children died of TB that year. Modelling studies also suggest that 750,000 adolescents (10 to < 20 years) develop TB disease each year globally [2]. Despite these numbers, child and adolescent TB remains an area generally neglected by research and programmatic prioritisation. Moreover, the Coronavirus Disease 2019 (COVID-19) pandemic caused by Severe Acute Respiratory Syndrome-Coronavirus-2 (SARS-CoV-2) has had a substantial impact on the global TB epidemic. The pandemic response has led to the fragmentation of TB services (including BCG vaccination) in many countries and high COVID-19 caseloads have placed additional pressure on overburdened health services and resulted in weakened national TB programmes, resulting in a decline in people accessing TB care and treatment and a rise in estimated TB deaths globally [3].

COVID-19 and TB share similar bio-social determinants, and it is speculated that the link between these two diseases may be bi-directional [4]. Studies on *Mycobacterium tuberculosis* (*Mtb)/*SARS-CoV-2 co-infections in adults suggest that COVID-19 can occur either before, during or after TB disease diagnosis and that TB is associated with increased COVID-19 morbidity and mortality [5–8]. In children and adolescents, the association between pulmonary TB and past SARS-CoV-2 infection remains understudied. Severe COVID-19 is characterized by lymphopenia and in combination with the use of immunosuppressive medications, could potentially lead to a reduced immune response to *Mtb*-specific antigens. Furthermore, other viral infections such as Human Immunodeficiency Virus (HIV), influenza and measles have been described to lead to an increased risk of TB disease in children and adults by either inducing immunosuppression or by disrupting mucosal integrity and altering host immunology [9, 10]. Both SARS-CoV-2 and *Mtb* principally affect the respiratory system and can elicit a hyperinflammatory state in the lung. It is therefore possible that the hyperinflammatory environment, induced by SARS-CoV-2 infection, could potentially accelerate TB disease progression [11]. Indirect evidence from a recent large global cohort, which included some children and adolescents, suggests that COVID-19 may not play a major role in facilitating the progression from *Mtb* infection to TB disease [12]. In contrast, a recent study from South Africa showed a significantly reduced frequency of *Mtb*-specific CD4 T cells in the peripheral blood of individuals with COVID-19, which supports the hypothesis that COVID-19 might increase the progression to TB disease in those with latent infection [13].

Despite attempts to elucidate interactions between SARS-CoV-2 and *Mtb*, several uncertainties still remain. Many individuals in high TB burden settings have viable and contained *Mtb* infection but are asymptomatic [14]. Improved characterisation of both cell-mediated and humoral immune responses to SARS-CoV-2 infection, in those who subsequently developed TB disease, can assist in unravelling the interplay between these two diseases and may help to identify those who are at greater risk of developing TB disease. We performed a case-control study to determine whether an association exists between previous SARS-CoV-2 infection and odds of pulmonary TB disease in children and adolescents from a high TB burden setting. We also evaluated the association between the magnitude of SARS-CoV-2 Immunoglobulin G (IgG) response and odds of pulmonary TB disease.

## Methods and materials

### Study design and setting

An unmatched case-control study was carried out to evaluate the association between past SARS-CoV-2 infection and odds of pulmonary TB disease in children and adolescents. The study utilised baseline clinical data and serum samples collected from the participants of two prospective cohort studies in Cape Town, South Africa. Cape Town is situated in the Western Cape province of South Africa and the overall TB incidence in the province was 681/100,000 in 2015 [15].

#### The Teen TB study

Teen TB aimed to better understand the biology, morbidity and social contexts of adolescent TB and how these interact. The study objectives were to evaluate the relationship between baseline imaging and respiratory function in adolescents with TB, to explore the psychosocial experience of adolescents affected by drug-susceptible and multidrug-resistant TB and to explore how pubertal hormones and viral co-infections influence the immune response to *Mtb.* The study included adolescents (aged 10 to < 20 years) with microbiologically confirmed pulmonary TB disease and healthy individuals exposed to an infectious case of pulmonary TB in their household. Clinical data collection, chest radiography, respiratory function assessment and blood sample collection were performed at baseline. Study methods and procedures for the Teen TB study are described in detail elsewhere [16].

#### The Umoya child TB study

The ongoing Umoya study is a TB diagnostic study that aims to develop a comprehensive clinical, radiological and biological biorepository to evaluate future diagnostic tools and biomarkers [17]. In addition, it aims to investigate long-term lung health outcomes. The study recruits children aged < 13 years, with HIV and without HIV, that present with well-defined symptoms suggestive of pulmonary TB from Tygerberg Children’s Hospital and Karl Bremer Hospital. These hospitals are regional referral centres and serve over 30% of the City of Cape Town metropolitan population. At baseline, a standard symptomatology questionnaire is completed, and a thorough physical examination is performed. A minimum of two respiratory samples are collected for TB investigations including sputum smear microscopy, liquid culture and the molecular diagnostic test, Xpert Ultra (Cepheid, CA, U.S.A.). Chest imaging (plain film chest x-ray), tuberculin skin test (TST) and HIV antibody testing are also done at baseline. The study includes children with TB (confirmed and unconfirmed); children in which TB was ruled out after careful investigations and follow-up (symptomatic controls) and asymptomatic sibling controls. Serum samples are collected as part of the biorepository and stored for later analysis.

### Study participants and sampling

The study population comprised individuals younger than 20 years of age who were recruited into the Teen TB and Umoya studies between 1 November 2020 and 1 November 2021 with available baseline demographic, clinical, laboratory and imaging data.

Individuals with TB (cases) and those without TB (controls) for the Teen TB and Umoya studies were defined as shown in Table 1. For our study, participants were classified as cases if a primary diagnosis of newly diagnosed pulmonary TB, with or without HIV co-infection, was made in a hospital or clinic and patients were within the first 14 days since diagnosis. Controls were defined as individuals younger than 20 years of age from similar epidemiological contexts as TB cases who were evaluated closely and found to not have current TB disease.

**Table 1:** Inclusion and exclusion criteria for Umoya and Teen TB studies

		Inclusion	Exclusion
**Umoya**	**Cases**	Any child aged < 13 years identified in hospital (inpatient or outpatient) with suspected pulmonary TB who: • Meets the criteria for confirmed TB or unconfirmed TB based on recent consensus agreement [18]	• Receipt of TB treatment for more than two days in the previous 14 days • Severe illness resulting in unstable condition • Any condition which would constitute an absolute contra-indication to any of the sampling procedures required by the study • Residence in remote areas with no ready access to transport for follow-up visits • Presence of only extra-thoracic TB without evidence of pulmonary TB
	**Controls** ***(asymptomatic)***	• Asymptomatic siblings of children enrolled with suspected pulmonary TB	• Severe illness resulting in unstable condition • Any condition which would constitute an absolute contra-indication to any of the sampling procedures required by the study
	**Controls** ***(symptomatic)***	• Symptomatic children who were evaluated in hospital and met the criteria for unlikely TB	
**Teen TB**	**Cases**	Any adolescent (10 to < 20 years) who has: • A primary diagnosis of newly diagnosed pulmonary TB bacteriologically confirmed on sputum (Xpert- or culture-positive), with or without HIV coinfection • And are within the first 14 days since diagnosis and thus 14 days of TB treatment	• Extrapulmonary TB without evidence of pulmonary TB • Severe illness or any condition causing the participant to be clinically unstable or require intensive care treatment • Pregnancy or breastfeeding • Diabetes Mellitus • Participants declining HIV testing for whom a recent (< 12 month) HIV test result is not available
	**Controls**	Any adolescent (10 to < 20 years): • Exposed in their household in the last 6 months to a case of infectious pulmonary TB • Has no symptoms of TB	• Previous TB disease • Severe illness or any condition causing the participant to be clinically unstable or require intensive care treatment • Pregnancy or breastfeeding • Diabetes Mellitus • Participants declining HIV testing for whom a recent (< 12 month) HIV test result is not available

Abbreviations: HIV, Human Immunodeficiency Virus; TB, Tuberculosis

### Laboratory analyses

Previous SARS-CoV-2 infection was defined as the detection of SARS-CoV-2 IgG antibodies in stored baseline serum of COVID-19 unvaccinated individuals. We also measured the magnitude of SARS-CoV-2 IgG response, using the value of the antibody titre. Baseline serum was stored as aliquots in 500 µl tubes at -80 °C until use. The specimens were tested for IgG antibodies to the SARS-CoV-2 spike protein S1 receptor-binding domain using the Abbott SARS-CoV-2 IgG II Quant chemiluminescent microparticle immunoassay (Abbott, IL, U.S.A.) on the Architect i System (Abbott). Laboratory staff from the Division of Medical Virology at Stellenbosch University (SU) performed the serological assay according to the manufacturer’s protocols, blinded to clinical characteristics. The default unit for the Abbott SARS-CoV-2 IgG II Quant assay is AU/ml; AU/ml values ≥ 50 and < 50 were defined as positive and negative, respectively, according to the manufacturer’s instructions. All tested samples were collected prior to SARS-CoV-2 vaccination roll-out for individuals aged under 18 years in South Africa.

### Statistical analysis

The number of eligible child and adolescent pulmonary TB cases and controls from the Teen TB and Umoya studies, who were enrolled during the study period, determined the study population for this hypothesis generating case-control study.

Data were analysed using STATA (version 17 STATA Corp., College Station, TX, USA). Descriptive analysis was used to characterise the study population, to compare the case and control groups and to aid in identifying differences between groups with respect to potential confounders.

Univariable logistic regression was performed to calculate unadjusted odds ratios (ORs) and accompanying 95% confidence intervals (CIs) for each covariable. The Teen TB and Umoya datasets were analysed separately before analysis of the combined dataset was performed. A forwards modelling approach was utilised to determine the final multivariable model for the combined Teen TB and Umoya dataset with age and sex included in the model *a priori*. For SARS-CoV-2 IgG seropositive samples, boxplots were generated to present the distribution of log-transformed viral-specific IgG response values for case and control groups. A Mann-Whitney U-test was used to assess whether the distribution of SARS-CoV-2 IgG values differed between case and control groups. Associations between SARS-CoV-2 IgG levels (tertiles) and pulmonary TB disease, adjusted for age, were also investigated further using an unconditional logistic regression model. Additional analyses of the Teen TB, Umoya and combined dataset were performed using different combinations of controls (**see Additional file 1**).

## Results

### Participants

**Fig. 1:**
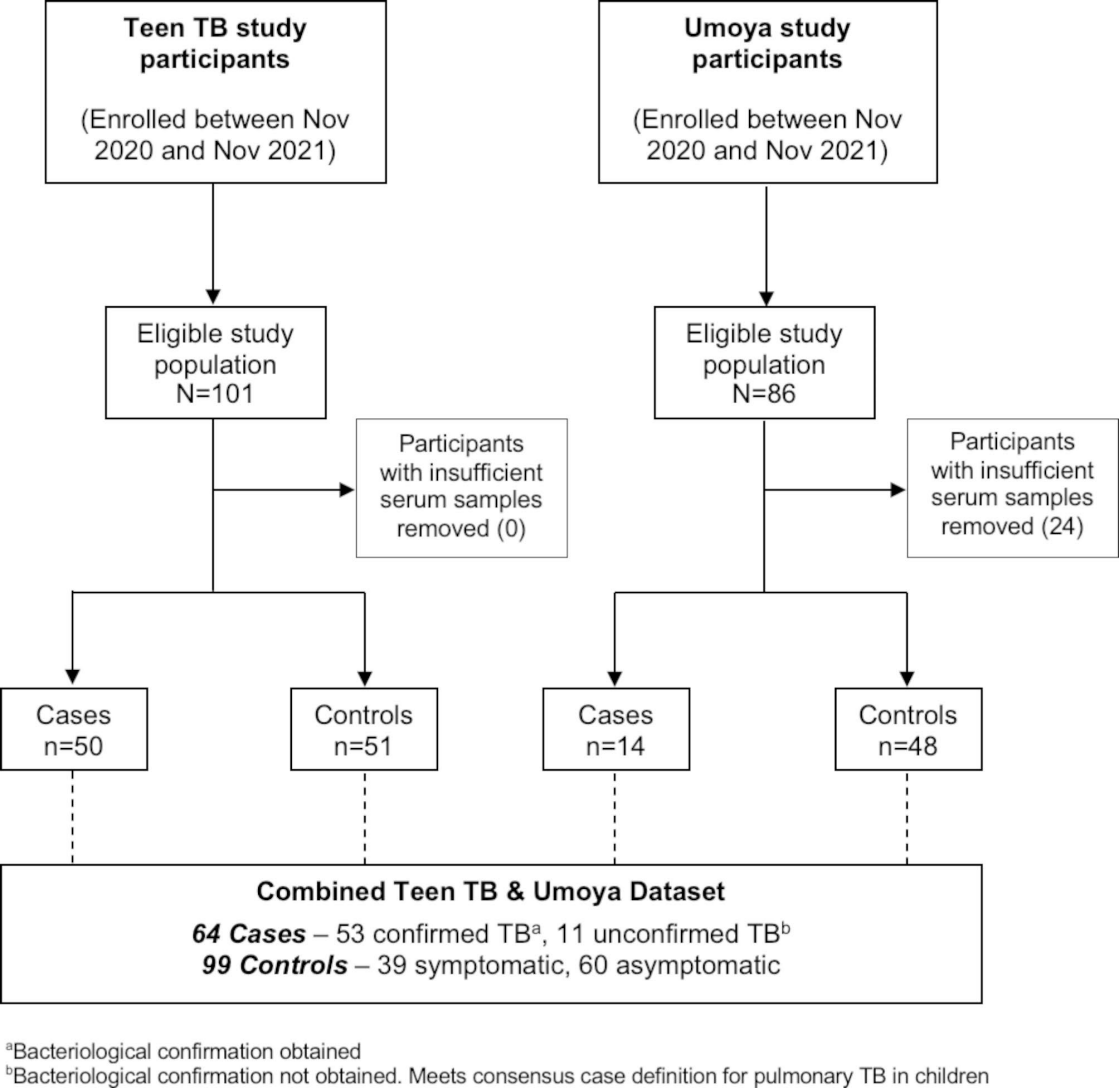
Flow-diagram of individuals included in the case-control study

One-hundred-and-one adolescents (10 to < 20 years of age) and 86 children (< 13 years of age) were enrolled into the Teen TB and Umoya studies between November 2020 and November 2021, respectively. Among the 86 eligible participants from the Umoya study, 24 children were excluded because insufficient volumes of stored baseline serum were available for SARS-CoV-2 IgG testing (Fig. 1).

The combined dataset included 163 participants, 53% (87/163) were female and the median age was 12 years (interquartile range [IQR] 3 to 16 years). Most children and adolescents were of mixed ancestry (51%; 83/163) and 32% (52/163) lived in informal housing. 9% (14/163) were people living with HIV and 47% (76/163) were SARS-CoV-2 IgG seropositive (Table 2, Supplementary Table S2).

**Table 2:** Baseline socio-demographic and clinical characteristics for each study and the combined dataset by case/ control group

	Teen TB		Umoya		Combined	
Characteristics	**Cases n (%)**	**Controls n (%)**	**Cases n (%)**	**Controls n (%)**	**Cases n (%)**	**Controls n (%)**
Overall	50	51	14	48	64	99
Age group (years)						
Under 5	-	-	13 (92.9)	42 (87.5)	13 (20.3)	42 (42.4)
5 to 9	-	-	1 (7.1)	6 (12.5)	1 (1.6)	6 (6.1)
10 to 14	9 (18.0)	33 (64.7)	-	-	9 (14.1)	33 (33.3)
15 to 19	41 (82.0)	18 (35.3)	-	-	41 (64.0)	18 (18.2)
Sex						
Male	18 (36.0)	28 (54.9)	5 (35.7)	25 (52.1)	23 (35.9)	53 (53.5)
Female	32 (64.0)	23 (45.1)	9 (64.3)	23 (47.9)	41 (64.1)	46 (46.5)
Ethnicity						
Black African	27 (54.0)	31 (60.8)	8 (57.1)	14 (29.2)	35 (54.7)	45 (45.5)
Mixed ancestry	23 (46.0)	20 (39.2)	6 (42.9)	34 (70.8)	29 (45.3)	54 (55.5)
Housing type						
Formal^a^	42 (84.0)	39 (76.5)	7 (50.0)	23 (47.9)	49 (76.6)	62 (62.6)
Informal^b^	8 (16.0)	12 (23.5)	7 (50.0)	25 (52.1)	15 (23.4)	37 (37.4)
Household size						
5 or less people	28 (56.0)	28 (54.9)	10 (71.4)	20 (41.7)	38 (59.4)	48 (48.5)
6 or more people	22 (44.0)	23 (45.1)	4 (28.6)	28 (58.3)	26 (40.6)	51 (51.5)
Cooking fuel						
Electricity or gas	49 (98.0)	49 (96.1)	14 (100.0)	46 (95.8)	63 (98.4)	95 (96.0)
Paraffin or coal	1 (2.0)	2 (3.9)	0 (0.0)	2 (4.2)	1 (1.6)	4 (4.0)
Water source						
Inside tap	42 (84.0)	42 (82.3)	10 (71.4)	32 (66.7)	52 (81.2)	74 (74.7)
Outside tap	8 (16.0)	9 (17.7)	4 (28.6)	16 (33.3)	12 (18.8)	25 (25.3)
Toilet location						
Inside house	42 (84.0)	37 (72.5)	8 (57.1)	32 (66.7)	50 (78.8)	69 (69.7)
Outside house	8 (16.0)	14 (27.5)	6 (42.9)	14 (29.2)	14 (21.9)	28 (28.3)
Missing	0 (0.0)	0 (0.0)	0 (0.0)	2 (4.1)	0 (0.0)	2 (2.0)
Primary caregiver						
Parent	43 (86.0)	47 (92.2)	10 (100.0)	42 (87.5)	57 (89.1)	89 (89.9)
Non-parent^c^	7 (14.0)	4 (7.8)	0 (0.0)	6 (12.5)	7 (10.9)	10 (10.1)
Anyone employed in house						
No	10 (20.0)	6 (11.8)	4 (28.6)	20 (41.7)	14 (21.9)	26 (26.3)
Yes	40 (80.0)	45 (88.2)	10 (71.4)	28 (58.3)	50 (78.1)	73 (73.7)
Household smoking exposure^d^						
No	19 (38.0)	13 (25.5)	7 (50.0)	18 (37.5)	26 (40.6)	31 (31.3)
Yes	31 (62.0)	38 (74.5)	7 (50.0)	30 (62.5)	38 (59.4)	68 (68.7)
Current smoker						
No	32 (64.0)	45 (88.2)	-	-	-	-
Yes	18 (36.0)	6 (11.8)	-	-	-	-
TB signs & symptoms						
Cough	42 (84.0)	2 (3.9)	9 (64.3)	33 (68.8)	51 (79.7)	35 (35.4)
Wheeze	20 (40.0)	0 (0.0)	4 (28.6)	10 (20.8)	24 (35.5)	10 (10.1)
Fever	7 (14.0)	0 (0.0)	5 (35.7)	19 (39.6)	12 (18.8)	19 (19.2)
Lack of appetite	21 (42.0)	0 (0.0)	7 (50.0)	14 (29.2)	28 (43.8)	14 (14.1)
Weight loss	39 (78.0)	1 (2.0)	-	-	39 (60.9)	1 (1.0)
Night sweats	29 (58.0)	0 (0.0)	-	-	29 (45.0)	0 (0.0)
Lymphadenopathy	4 (8.0)	1 (2.0)	1 (7.1)	2 (4.2)	5 (7.8)	3 (3.0)
Chronic lung disease signs^e^						
No	49 (98.0)	51 (100.0)	13 (92.9)	46 (95.8)	62 (96.9)	97 (98.0)
Yes	1 (2.0)	0 (0.0)	1 (7.1)	2 (4.2)	2 (3.1)	2 (2.0)
BCG scar						
No	0 (0.0)	1 (2.0)	2 (14.3)	9 (18.8)	2 (3.1)	10 (10.1)
Yes	49 (98.0)	49 (96.0)	11 (78.6)	39 (81.2)	60 (93.8)	88 (88.9)
Missing	1 (2.0)	1 (2.0)	1 (7.1)	0 (0.0)	2 (3.1)	1 (1.0)
Previous TB disease						
No	41 (82.0)	51 (100.0)	12 (85.7)	39 (81.2)	53 (82.9)	90 (90.9)
Yes	9 (18.0)	0 (0.0)	2 (14.3)	9 (18.8)	11 (17.2)	9 (9.1)
HIV status						
Negative	45 (90.0)	50 (98.0)	11 (78.6)	43 (89.6)	56 (87.5)	93 (93.9)
Positive	5 (10.0)	1 (2.0)	3 (21.4)	5 (10.4)	8 (12.5)	6 (6.1)
SARS-CoV-2 IgG serostatus						
Negative	27 (54.0)	20 (39.2)	10 (71.4)	30 (62.5)	37 (57.8)	50 (50.5)
Positive	23 (46.0)	31 (60.8)	4 (28.6)	18 (37.5)	27 (42.2)	49 (49.5)

Abbreviations: BCG, Bacillus Calmette-Guérin; IgG, Immunoglobulin G; SARS-CoV-2, Severe Acute Respiratory Syndrome – Coronavirus – 2; TB, Tuberculosis. ^a^Formal: Brick house, ^b^Informal: Wendy house or shack, ^c^Non-parent: Grandmother/ father, other family or community member, ^d^Household smoking exposure: Exposure to second-hand tobacco smoke in the household, ^e^Chronic lung disease signs: Chest deformity, clubbing, coarse crackles or pulmonary hypertension

### SARS-CoV-2 IgG serostatus and risk of pulmonary TB

There was no significant difference in the odds of pulmonary TB disease between those with positive and negative SARS-CoV-2 IgG serology in the combined dataset (unadjusted OR 0.74 95% CI: 0.40–1.40; p = 0.36) (Table 3). After adjusting for age group (four levels), sex and household size, there was no statistically significant difference in the odds of pulmonary TB disease among those who were SARS-CoV-2 IgG seropositive compared to those who were SARS-CoV-2 IgG seronegative (adjusted OR 0.51 95% CI: 0.23–1.11; n = 163, p = 0.09). There was no evidence of an interaction between SARS-CoV-2 IgG serostatus and age group (p = 0.81) or sex (p = 0.32) in the combined dataset.

**Table 3:** Unadjusted and adjusted estimates of the association between SARS-CoV-2 IgG serostatus and pulmonary TB disease for each study and the combined dataset

Dataset	Participant group	SARS-CoV-2 IgG seropositive/ seronegative	Unadjusted OR (95% CI)	p-value	Adjusted OR^a^ (95% CI)	p-value
Teen TB	Non-TB controls	31/20	1.0		1.0	
	TB cases	23/27	0.55 (0.25–1.21)	0.14	0.61 (0.21–1.77)	0.84
Umoya	Non-TB controls	18/30	1.0		-	
	TB cases	4/10	0.67 (0.18–2.44)	0.54	-	-
Combined	Non-TB controls	49/50	1.0		1.0	
	TB cases	27/37	0.74 (0.40–1.40)	0.36	0.51 (0.23–1.11)	0.09

Abbreviations: CI, Confidence Interval; IgG, Immunoglobulin G; OR, Odds Ratio; SARS-CoV-2, Severe Acute Respiratory Syndrome-Coronavirus-2; TB, Tuberculosis

^a^Adjusted for age, sex, anyone employed in house and housing category in the Teen TB dataset. Adjusted for age group, sex and household size category in the Combined dataset

All p-values calculated from a likelihood ratio test

### SARS-CoV-2 IgG response and risk of pulmonary TB

**Fig. 2:**
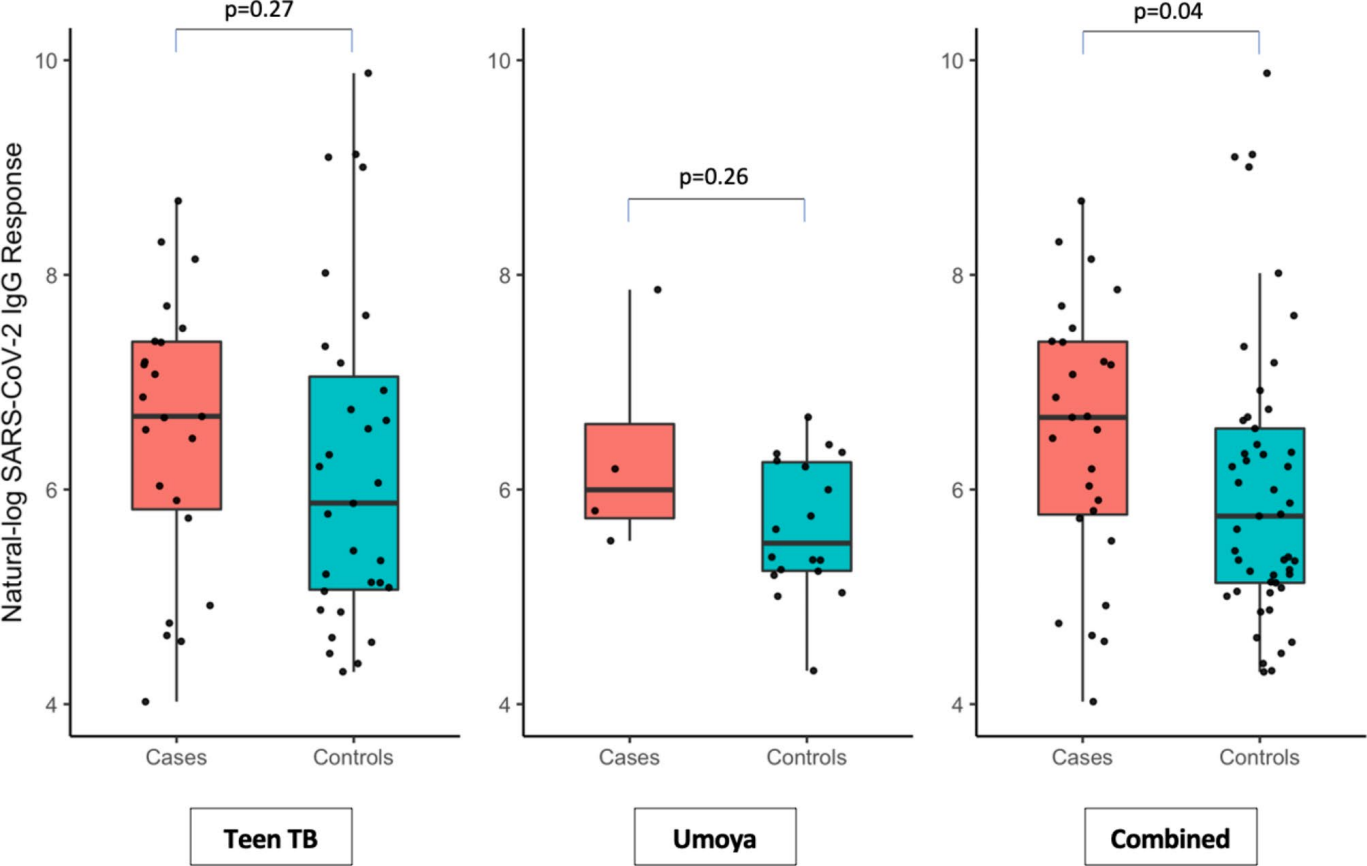
Log-transformed SARS-CoV-2 IgG response values for SARS-CoV-2 IgG positive cases and controls with accompanying p-value from a Mann-Whitney U-test

In the combined dataset, TB cases who were SARS-CoV-2 IgG seropositive had a median IgG value of 790 AU/ml (IQR 308 to 1605) and seropositive controls had a median IgG value of 315 AU/ml (IQR 169 to 712; p = 0.04) (Fig. 2). We did not find evidence that median IgG values differed with age group (under 10 and 10 to < 20 years) (p = 0.26) or that median values differed with sex (p = 0.09).

SARS-CoV-2 IgG values in the upper tertile of the range were associated with 4 times greater odds of having pulmonary TB disease compared with low IgG levels (95% CI: 1.13–14.21; p = 0.03; Table 4). We found evidence for the directional trend to increased risk of pulmonary TB disease with increasing SARS-CoV-2 IgG levels (p = 0.01). There was no evidence for a departure from linearity (p = 0.80).

**Table 4:** Odds of pulmonary TB by SARS-CoV-2 immunoglobulin G levels^a^

Viral-specific IgG level	Number of serum samples	Adjusted OR^b^ (95% CI)	p-value for trend^c^
SARS-CoV-2			
Low (55.90–209.49 AU/ml)	25	1.0 (Reference)	0.01
Medium (209.50–767.39 AU/ml)	25	2.48 (0.63–9.71)	
High (767.40–19529.90 AU/ml)	26	4.00 (1.13–14.21)	

Abbreviations: AU, Arbitrary Units; CI, Confidence Interval; IgG, Immunoglobulin G; OR, Odds Ratio; SARS-CoV-2, Severe Acute Respiratory – Coronavirus – 2

^a^Medium and high tertiles are compared with the lowest tertile of IgG level in an unconditional logistic regression model

^b^Adjusted for age group only in SARS-CoV-2 model

^c^P value from a likelihood ratio test for trend

## Discussion

We hypothesised that SARS-CoV-2 IgG seropositivity, as a marker of past infection, was associated with an increased odds of pulmonary TB disease in children and adolescents. After combining both Teen TB and Umoya datasets and adjusting for age group, sex and household size, we did not find convincing evidence of a relationship between previous SARS-CoV-2 infection and pulmonary TB disease. However, using IgG antibody response to the SARS-CoV-2 spike protein S1 receptor-binding domain, we showed that the magnitude of serological response to SARS-CoV-2 amongst those with serological evidence of previous infection at baseline was associated with an increased odds of pulmonary TB disease, in a dose-response manner.

To our knowledge, this is the first study to evaluate the association between past SARS-CoV-2 infection and pulmonary TB disease in children and adolescents. Our findings could, in large part, be explained by the biases associated with the types of controls selected, which likely resulted in more controls having the exposure of interest compared to cases. It is possible that hospital-based symptomatic controls from the Umoya study may have had a recent SARS-CoV-2 infection that contributed towards their need for hospital admission. Furthermore, crowded health facilities are high risk settings for acquiring respiratory infections and frequent visits to health facilities by symptomatic controls, prior to hospital admission, could have increased their risk of acquiring SARS-CoV-2. Almost half of the controls from the Teen TB study were recruited to the study later than cases; therefore, the local seroprevalence of SARS-CoV-2 at the time of control recruitment could likely have been higher compared to when cases were recruited. This assumption is supported by recent seroprevalence data from Cape Town, which showed that SARS-CoV-2 anti-nucleocapsid seropositivity increased from ~ 39% in August 2020 to almost 68% in November 2021 [19]. However, when we included recruitment period (by quarter) in the regression model there was little, if any, confounding. Additionally, a recent study from India found that adults with *Mtb* infection and previous SARS-CoV-2 infection exhibited increased SARS-CoV-2 IgG levels and enhanced neutralising antibody activity compared to adults without *Mtb* infection [20]. Given that more than 75% of adolescent Teen TB controls were Interferon Gamma Release Assay (IGRA)-positive, increased IgG concentrations could potentially have increased the chances of these adolescents having positive SARS-CoV-2 IgG serology. Despite having more IgG seropositive child and adolescent controls in this study and some adolescents being more likely to have higher IgG concentrations (*Mtb* infection), increasing SARS-CoV-2 IgG levels were still associated with increased odds of pulmonary TB disease after adjusting for age. However, it was not possible to adjust for sex or HIV status due to the small number of cases in the model.

While our study does not address cellular immunity, it allows for indirect inferences about the T helper (Th)-2 effector response, because a strong SARS-CoV-2 IgG response relies on adequate Th-2 effector activation [21]. A strong Th-1 response is known to protect against the development of TB disease [22, 23]. Similarly, a coordinated Th-1 immune response to SARS-CoV-2 is associated with a good prognosis and resolution of COVID-19 in adults while Th-1 hypoactivation and Th-2 overreaction, with subsequent exhaustion, has been found to be associated with a worse prognosis [24]. Younger children, which have a high risk of progressing from *Mtb* infection to TB disease, also have poorly functioning innate cells and a Th-2 skew [25]. Kaiko and colleagues found that when a Th effector response is polarized to Th-2, antibody production is not only stimulated but the cell-mediated immunity is also suppressed [26]. The above mechanism could explain why an increasing SARS-CoV-2 IgG response is associated with increased odds of pulmonary TB disease.

In our study, the timing of a previous SARS-CoV-2 infection and the severity of prior COVID-19 were not known for those who tested SARS-CoV-2 IgG positive. More recent infection and more severe COVID-19 have been found to trigger extensive humoral responses and can result in higher IgG titres [27, 28]. Asymptomatic SARS-CoV-2 infections likely elicit a weaker antibody response [29] and the time course and duration of humoral immune responses are potentially very different in asymptomatic SARS-CoV-2 infections [30, 31]. The absence of this information makes the interpretation of our SARS-CoV-2 IgG response findings challenging. Moreover, individuals with mild COVID-19 symptoms may have a robust mucosal immune response within the respiratory tract that controls the virus and a limited systemic immune response [32].

A strength of the study includes the use of a serology testing strategy that allows for the interpretation of an exposure-outcome temporal association. Testing serum samples from individuals with TB who were within the first 14-day since diagnosis limited the chance of detecting IgG antibodies against SARS-CoV-2 that could have been acquired after the diagnosis of pulmonary TB disease was made. However, the possibility of TB disease preceding the SARS-CoV-2 infection still exists. Another strength is the inclusion of more controls than TB cases that come from the same cohort population. Our study does also have several limitations. The study was inadequately powered to investigate the main study associations and random error cannot be excluded. The Teen TB and Umoya studies provided different control groups for our study, and it is likely that the background frequency of SARS-CoV-2 IgG seropositivity differed between control groups and the general population; therefore, the risk of selection bias was high. There is also a risk of residual confounding in this study. SARS-CoV-2 and TB share similar bio-social determinants [33] and there are likely many potential confounders that need to be considered when investigating associations between these two infections. Furthermore, potential confounders such as sex and HIV status could not be included in the model examining the association between SARS-CoV-2 IgG tertiles and TB disease due to the small number of IgG seropositive individuals with TB disease. The matching of cases and controls on key confounders such as age and sex was not possible in this study but would likely have benefitted our overall analysis and enabled adjustment for difficult to measure confounding variables (e.g., household matching). The study included children and adolescents from a high TB-burden setting in South Africa; therefore, findings can, to some extent, be generalised to other high TB-burden settings. Given that approximately one quarter of the world’s population is estimated to be infected with *Mtb* [14] and the ongoing nature of the SARS-CoV-2 pandemic, findings are likely generalisable to more settings. However, the grouping into SARS-CoV-2 IgG tertiles was based on IgG ranges found in our population and may not be generalisable to other populations with different SARS-CoV-2 transmission dynamics.

Improved characterisation of the shared dysregulation of immunological responses in COVID-19 and TB in blood and lung tissue will help to determine whether more severe SARS-CoV-2 infection/ COVID-19 is a risk factor for progression to TB disease or increases susceptibility to infection. For children and adolescents, large longitudinal cohorts evaluating how host immunological responses to SARS-CoV-2 and *Mtb* change with age, sex and puberty would also be informative.

Our study is the first study in Africa to evaluate the association between the magnitude of SARS-CoV-2-specific IgG responses and odds of pulmonary TB disease in children and adolescents and adds to the growing body of knowledge on the association between these two infections.

## Electronic supplementary material

Below is the link to the electronic supplementary material.

Supplementary Material 1

## Data Availability

The datasets used and/or analysed during the current study are available from the corresponding author on reasonable request. ***Competing interests***. The authors declare that they have no competing interests.

## Abbreviations

AU: Arbitrary Units
BCG: Bacillus Calmette–Guérin
CI: Confidence Interval
COVID-19: Coronavirus Disease 2019
HIV: Human Immunodeficiency Virus
IgG: Immunoglobulin G
IQR: Interquartile Range
IGRA: Interferon Gamma Release Assay
LRT: Likelihood Ratio Test
LSHTM: London School of Hygiene and Tropical Medicine
Mtb: Mycobacterium Tuberculosis
NHLS: National Health Laboratory Service
OR: Odds Ratio
SARS-CoV-2: Severe Acute Respiratory Syndrome-Coronavirus-2
SU: Stellenbosch University
TB: Tuberculosis
Th: T helper
TST: Tuberculin Skin Test
WHO: World Health Organization
