# Supplementary material for: SARS-CoV-2 infection and pulmonary tuberculosis in children and adolescents: a case-control study
Source: BMC Infect Dis. 2023 Jun 29;23:442. doi: 10.1186/s12879-023-08412-8 (PMC10311805; doi:10.1186/s12879-023-08412-8)
Supplement: Supplementary file 1 — Supplementary Material 1 [file 12879_2023_8412_MOESM1_ESM.docx]

**ADDITIONAL FILE**

**Manuscript Title**

SARS-CoV-2 infection and pulmonary tuberculosis in children and adolescents: A case-control study

**Authors**

Jeremi Swanepoel^1,2^

Marieke M. van der Zalm^1^

Wolfgang Preiser^3^

Gert van Zyl^3^

Elizabeth Whittaker^4^

Anneke C. Hesseling^1^

David A. J Moore^2^

James A. Seddon^1,4^

**Affiliations**

^1^Desmond Tutu TB Centre, Department of Paediatrics and Child Health, Faculty of Medicine and Health Sciences, Stellenbosch University, South Africa

^2^ TB Centre, London School of Hygiene and Tropical Medicine, London, United Kingdom

^3^Division of Medical Virology, Department of Pathology, Faculty of Medicine and Health Sciences, Stellenbosch University and National Health Laboratory Service, Tygerberg Academic Hospital, Cape Town, South Africa

^4^Department of Infectious Disease, Imperial College London, London, United Kingdom

**Supplementary Table S2:** Baseline socio-demographic and clinical characteristics for each study and the combined dataset by case/ control group.

|  | Teen TB | | Umoya | | Combined | |  |
| --- | --- | --- | --- | --- | --- | --- | --- |
| Characteristics | **Cases n (%)** | **Controls n (%)** | **Cases n (%)** | **Controls n (%)** | **Cases n (%)** | **Controls n (%)** | **p-value*** |
| Overall | 50 | 51 | 14 | 48 | 64 | 99 |  |
| Age group (years) |  |  |  |  |  |  | <0.001 |
| Under 5 | - | - | 13 (92.9) | 42 (87.5) | 13 (20.3) | 42 (42.4) |  |
| 5 to 9 | - | - | 1 (7.1) | 6 (12.5) | 1 (1.6) | 6 (6.1) |  |
| 10 to 14 | 9 (18.0) | 33 (64.7) | - | - | 9 (14.1) | 33 (33.3) |  |
| 15 to 19 | 41 (82.0) | 18 (35.3) | - | - | 41 (64.0) | 18 (18.2) |  |
| Sex |  |  |  |  |  |  | 0.03 |
| Male | 18 (36.0) | 28 (54.9) | 5 (35.7) | 25 (52.1) | 23 (35.9) | 53 (53.5) |  |
| Female | 32 (64.0) | 23 (45.1) | 9 (64.3) | 23 (47.9) | 41 (64.1) | 46 (46.5) |  |
| Ethnicity |  |  |  |  |  |  | 0.25 |
| Black African | 27 (54.0) | 31 (60.8) | 8 (57.1) | 14 (29.2) | 35 (54.7) | 45 (45.5) |  |
| Mixed ancestry | 23 (46.0) | 20 (39.2) | 6 (42.9) | 34 (70.8) | 29 (45.3) | 54 (55.5) |  |
| Housing type |  |  |  |  |  |  |  |
| Formal^a^ | 42 (84.0) | 39 (76.5) | 7 (50.0) | 23 (47.9) | 49 (76.6) | 62 (62.6) |  |
| Informal^b^ | 8 (16.0) | 12 (23.5) | 7 (50.0) | 25 (52.1) | 15 (23.4) | 37 (37.4) |  |
| Household size |  |  |  |  |  |  | 0.17 |
| 5 or less people | 28 (56.0) | 28 (54.9) | 10 (71.4) | 20 (41.7) | 38 (59.4) | 48 (48.5) |  |
| 6 or more people | 22 (44.0) | 23 (45.1) | 4 (28.6) | 28 (58.3) | 26 (40.6) | 51 (51.5) |  |
| Cooking fuel |  |  |  |  |  |  | 0.37 |
| Electricity or gas | 49 (98.0) | 49 (96.1) | 14 (100.0) | 46 (95.8) | 63 (98.4) | 95 (96.0) |  |
| Paraffin or coal | 1 (2.0) | 2 (3.9) | 0 (0.0) | 2 (4.2) | 1 (1.6) | 4 (4.0) |  |
| Water source |  |  |  |  |  |  | 0.33 |
| Inside tap | 42 (84.0) | 42 (82.3) | 10 (71.4) | 32 (66.7) | 52 (81.2) | 74 (74.7) |  |
| Outside tap | 8 (16.0) | 9 (17.7) | 4 (28.6) | 16 (33.3) | 12 (18.8) | 25 (25.3) |  |
| Toilet location |  |  |  |  |  |  | 0.38 |
| Inside house | 42 (84.0) | 37 (72.5) | 8 (57.1) | 32 (66.7) | 50 (78.8) | 69 (69.7) |  |
| Outside house | 8 (16.0) | 14 (27.5) | 6 (42.9) | 14 (29.2) | 14 (21.9) | 28 (28.3) |  |
| Missing | 0 (0.0) | 0 (0.0) | 0 (0.0) | 2 (4.1) | 0 (0.0) | 2 (2.0) |  |
| Primary caregiver |  |  |  |  |  |  | 0.87 |
| Parent | 43 (86.0) | 47 (92.2) | 10 (100.0) | 42 (87.5) | 57 (89.1) | 89 (89.9) |  |
| Non-parent^c^ | 7 (14.0) | 4 (7.8) | 0 (0.0) | 6 (12.5) | 7 (10.9) | 10 (10.1) |  |
| Anyone employed in house |  |  |  |  |  |  | 0.53 |
| No | 10 (20.0) | 6 (11.8) | 4 (28.6) | 20 (41.7) | 14 (21.9) | 26 (26.3) |  |
| Yes | 40 (80.0) | 45 (88.2) | 10 (71.4) | 28 (58.3) | 50 (78.1) | 73 (73.7) |  |
| Household smoking exposure^d^ |  |  |  |  |  |  | 0.22 |
| No | 19 (38.0) | 13 (25.5) | 7 (50.0) | 18 (37.5) | 26 (40.6) | 31 (31.3) |  |
| Yes | 31 (62.0) | 38 (74.5) | 7 (50.0) | 30 (62.5) | 38 (59.4) | 68 (68.7) |  |
| Current smoker |  |  |  |  |  |  | - |
| No | 32 (64.0) | 45 (88.2) | - | - | - | - |  |
| Yes | 18 (36.0) | 6 (11.8) | - | - | - | - |  |
| TB signs & symptoms |  |  |  |  |  |  |  |
| Cough | 42 (84.0) | 2 (3.9) | 9 (64.3) | 33 (68.8) | 51 (79.7) | 35 (35.4) | <0.001 |
| Wheeze | 20 (40.0) | 0 (0.0) | 4 (28.6) | 10 (20.8) | 24 (35.5) | 10 (10.1) | <0.001 |
| Fever | 7 (14.0) | 0 (0.0) | 5 (35.7) | 19 (39.6) | 12 (18.8) | 19 (19.2) | 0.94 |
| Lack of appetite | 21 (42.0) | 0 (0.0) | 7 (50.0) | 14 (29.2) | 28 (43.8) | 14 (14.1) | <0.001 |
| Weight loss | 39 (78.0) | 1 (2.0) | - | - | 39 (60.9) | 1 (1.0) | <0.001 |
| Night sweats | 29 (58.0) | 0 (0.0) | - | - | 29 (45.0) | 0 (0.0) | <0.001 |
| Lymphadenopathy | 4 (8.0) | 1 (2.0) | 1 (7.1) | 2 (4.2) | 5 (7.8) | 3 (3.0) | 0.27 |
| Chronic lung disease signs^e^ |  |  |  |  |  |  | 0.65 |
| No | 49 (98.0) | 51 (100.0) | 13 (92.9) | 46 (95.8) | 62 (96.9) | 97 (98.0) |  |
| Yes | 1 (2.0) | 0 (0.0) | 1 (7.1) | 2 (4.2) | 2 (3.1) | 2 (2.0) |  |
| BCG scar |  |  |  |  |  |  | 0.13 |
| No | 0 (0.0) | 1 (2.0) | 2 (14.3) | 9 (18.8) | 2 (3.1) | 10 (10.1) |  |
| Yes | 49 (98.0) | 49 (96.0) | 11 (78.6) | 39 (81.2) | 60 (93.8) | 88 (88.9) |  |
| Missing | 1 (2.0) | 1 (2.0) | 1 (7.1) | 0 (0.0) | 2 (3.1) | 1 (1.0) |  |
| Previous TB disease |  |  |  |  |  |  | 0.12 |
| No | 41 (82.0) | 51 (100.0) | 12 (85.7) | 39 (81.2) | 53 (82.9) | 90 (90.9) |  |
| Yes | 9 (18.0) | 0 (0.0) | 2 (14.3) | 9 (18.8) | 11 (17.2) | 9 (9.1) |  |
| HIV status |  |  |  |  |  |  | 0.15 |
| Negative | 45 (90.0) | 50 (98.0) | 11 (78.6) | 43 (89.6) | 56 (87.5) | 93 (93.9) |  |
| Positive | 5 (10.0) | 1 (2.0) | 3 (21.4) | 5 (10.4) | 8 (12.5) | 6 (6.1) |  |
| SARS-CoV-2 IgG serostatus |  |  |  |  |  |  | 0.36 |
| Negative | 27 (54.0) | 20 (39.2) | 10 (71.4) | 30 (62.5) | 37 (57.8) | 50 (50.5) |  |
| Positive | 23 (46.0) | 31 (60.8) | 4 (28.6) | 18 (37.5) | 27 (42.2) | 49 (49.5) |  |
|  |  |  |  |  |  |  |  |

Abbreviations: BCG, Bacillus Calmette-Guérin; IgG, Immunoglobulin G; SARS-CoV-2, Severe Acute Respiratory Syndrome – Coronavirus – 2; TB, Tuberculosis. ^a^Formal: Brick house, ^b^Informal: Wendy house or shack, ^c^Non-parent: Grandmother/ father, other family or community member, ^d^Household smoking exposure: Exposure to second-hand tobacco smoke in the household , ^e^Chronic lung disease signs: Chest deformity, clubbing, coarse crackles or pulmonary hypertension

*Calculated using a Chi-square test for the combined dataset only

**Sensitivity Analysis 1:** All cases (n=64) and only asymptomatic controls (n=60) from the combined dataset.

| Table. Unadjusted and adjusted estimates of the association between SARS-CoV-2 IgG serostatus and pulmonary TB disease for each study and the combined dataset. | | | | | | |
| --- | --- | --- | --- | --- | --- | --- |
| Dataset | **Participant group** | **SARS-CoV-2 IgG seropositive/ seronegative** | **Unadjusted OR**  **(95% CI)** | **p-value** | **Adjusted OR^a^**  **(95% CI)** | **p-value** |
|  |  |  |  |  |  |  |
| Teen TB | Non-TB controls | 31/20 | 1.0 |  | 1.0 |  |
|  | TB cases | 23/27 | 0.55 (0.25 – 1.21) | 0.14 | 0.61 (0.21 0 1.77) | 0.84 |
|  |  |  |  |  |  |  |
|  |  |  |  |  |  |  |
| Umoya | Non-TB controls | 3/6 | 1.0 |  | - |  |
|  | TB cases | 4/10 | 0.80 (0.10 – 7.52) | 1.00 | - | - |
|  |  |  |  |  |  |  |
|  |  |  |  |  |  |  |
| Combined | Non-TB controls | 34/26 | 1.0 |  | 1.0 |  |
|  | TB cases | 27/37 | 0.56 (0.27 – 1.14) | 0.11 | 0.57 (0.25 – 1.30) | 0.18 |
|  |  |  |  |  |  |  |

Abbreviations: CI, Confidence Interval; IgG, Immunoglobulin G; OR, Odds Ratio; SARS-CoV-2, Severe Acute Respiratory Syndrome-Coronavirus-2; TB, Tuberculosis.

^a^Adjusted for age, sex, anyone employed in house and housing category in the Teen TB dataset. Adjusted for age group and sex in the Combined dataset.

All p-values calculated from a likelihood ratio test

| Table. Odds of pulmonary TB by SARS-CoV-2 immunoglobulin G levels^a^ | | | |
| --- | --- | --- | --- |
| Viral-specific IgG level | **Sample, No.** | **Adjusted OR^b^ (95% CI)** | **p-value for trend^c^** |
| SARS-CoV-2 |  |  |  |
| Low (55.90 – 227.70 AU/ml) | 20 | 1.0 (Reference) | 0.04 |
| Medium (227.80 – 951.99 AU/ml) | 20 | 3.65 (0.90 – 14.77) |  |
| High (952.00 – 19529.90 AU/ml) | 21 | 3.83 (0.98 – 14.89) |  |
|  |  |  |  |

Abbreviations: AU, Arbitrary Units; CI, Confidence Interval; IgG, Immunoglobulin G; OR, Odds Ratio; SARS-CoV-2, Severe Acute Respiratory – Coronavirus – 2.

^a^Medium and high tertiles are compared with the lowest tertile of IgG level in an unconditional logistic regression model.

^b^Adjusted for age only in SARS-CoV-2 model.

^c^P value from a likelihood ratio test for trend.

**Sensitivity Analysis 2:** All Teen TB cases (n=50) and controls (n=51) alone.

| Table. Unadjusted and adjusted estimates of the association between SARS-CoV-2 IgG serostatus and pulmonary TB disease for Teen TB. | | | | | | |
| --- | --- | --- | --- | --- | --- | --- |
| Dataset | **Participant group** | **SARS-CoV-2 IgG seropositive/ seronegative** | **Unadjusted OR**  **(95% CI)** | **p-value** | **Adjusted OR^a^**  **(95% CI)** | **p-value** |
|  |  |  |  |  |  |  |
| Teen TB | Non-TB controls | 31/20 | 1.0 |  | 1.0 |  |
|  | TB cases | 23/27 | 0.55 (0.25 – 1.21) | 0.14 | 0.61 (0.21 0 1.77) | 0.84 |
|  |  |  |  |  |  |  |

Abbreviations: CI, Confidence Interval; IgG, Immunoglobulin G; OR, Odds Ratio; SARS-CoV-2, Severe Acute Respiratory Syndrome-Coronavirus-2; TB, Tuberculosis.

^a^Adjusted for age, sex, anyone employed in house and housing category.

All p-values calculated from a likelihood ratio test

| Table. Odds of pulmonary TB by SARS-CoV-2 immunoglobulin G levels for Teen TB^a^ | | | |
| --- | --- | --- | --- |
| Viral-specific IgG level | **Sample, No.** | **Adjusted OR^b^ (95% CI)** | **p-value for trend^c^** |
| SARS-CoV-2 |  |  |  |
| Low (55.90 – 217.54 AU/ml) | 18 | 1.0 (Reference) | 0.09 |
| Medium (217.55 – 1096.39 AU/ml) | 18 | 3.47 (0.56 – 21.63) |  |
| High (1096.40 – 19529.90 AU/ml) | 18 | 3.88 (0.64 – 23.46) |  |
|  |  |  |  |

Abbreviations: AU, Arbitrary Units; CI, Confidence Interval; IgG, Immunoglobulin G; OR, Odds Ratio; SARS-CoV-2, Severe Acute Respiratory – Coronavirus – 2.

^a^Medium and high tertiles are compared with the lowest tertile of IgG level in an unconditional logistic regression model.

^b^Adjusted for age only in SARS-CoV-2 model.

^c^P value from a likelihood ratio test for trend.

**Sensitivity Analysis 3:** All Umoya cases (n=14) and controls (n=48) alone.

| Table. Unadjusted estimates of the association between SARS-CoV-2 IgG serostatus and pulmonary TB disease for the Umoya study. | | | | | | |
| --- | --- | --- | --- | --- | --- | --- |
| Dataset | **Participant group** | **SARS-CoV-2 IgG seropositive/ seronegative** | **Unadjusted OR**  **(95% CI)** | **p-value** | **Adjusted OR^a^**  **(95% CI)** | **p-value** |
|  |  |  |  |  |  |  |
| Umoya | Non-TB controls | 18/30 | 1.0 |  | - |  |
|  | TB cases | 4/10 | 0.67 (0.18 – 2.44) | 0.54 | - | - |
|  |  |  |  |  |  |  |

Abbreviations: CI, Confidence Interval; IgG, Immunoglobulin G; OR, Odds Ratio; SARS-CoV-2, Severe Acute Respiratory Syndrome-Coronavirus-2; TB, Tuberculosis.

^a^Adjusted OR not calculated due to too few outcome events per predictor variable.

All p-values calculated from a likelihood ratio test

| Table. Odds of pulmonary TB by SARS-CoV-2 immunoglobulin G levels^a^ | | | |
| --- | --- | --- | --- |
| Viral-specific IgG level | **Sample, No.** | **Unadjusted OR (95% CI)** | **p-value for trend^b^** |
| SARS-CoV-2 |  |  |  |
| Low (74.50 – 209.49 AU/ml) | 7 | 1.0 (Reference) | 0.21 |
| Medium (209.50 – 488.69 AU/ml) | 7 | 1.20 (0.12 – 11.87) |  |
| High (488.70 – 2598.40 AU/ml) | 8 | 1.00 (Omitted) |  |
|  |  |  |  |

Abbreviations: AU, Arbitrary Units; CI, Confidence Interval; IgG, Immunoglobulin G; OR, Odds Ratio; SARS-CoV-2, Severe Acute Respiratory – Coronavirus – 2.

^a^Medium and high tertiles are compared with the lowest tertile of IgG level in an unconditional logistic regression model.

^b^P value from a likelihood ratio test for trend.

**Sensitivity Analysis 4:** All Umoya cases (n=14) and symptomatic controls (n=39)

| Table. Unadjusted estimates of the association between SARS-CoV-2 IgG serostatus and pulmonary TB disease for the Umoya study. | | | | | | |
| --- | --- | --- | --- | --- | --- | --- |
| Dataset | **Participant group** | **SARS-CoV-2 IgG seropositive/ seronegative** | **Unadjusted OR**  **(95% CI)** | **p-value** | **Adjusted OR^a^**  **(95% CI)** | **p-value** |
|  |  |  |  |  |  |  |
| Umoya | Non-TB controls | 15/24 | 1.0 |  | - |  |
|  | TB cases | 4/10 | 0.64 (0.17 – 2.41) | 0.51 | - | - |
|  |  |  |  |  |  |  |

Abbreviations: CI, Confidence Interval; IgG, Immunoglobulin G; OR, Odds Ratio; SARS-CoV-2, Severe Acute Respiratory Syndrome-Coronavirus-2; TB, Tuberculosis.

^a^Adjusted OR not calculated due to too few outcome events per predictor variable.

All p-values calculated from a likelihood ratio test

| Table. Odds of pulmonary TB by SARS-CoV-2 immunoglobulin G levels^a^ | | | |
| --- | --- | --- | --- |
| Viral-specific IgG level | **Sample, No.** | **Unadjusted OR (95% CI)** | **p-value for trend^b^** |
| SARS-CoV-2 |  |  |  |
| Low (74.50 – 209.49 AU/ml) | 6 | 1.0 (Reference) | 0.21 |
| Medium (209.50 – 488.69 AU/ml) | 6 | 1.25 (0.12 – 13.24) |  |
| High (488.70 – 2598.40 AU/ml) | 7 | 1.00 (Omitted) |  |
|  |  |  |  |

Abbreviations: AU, Arbitrary Units; CI, Confidence Interval; IgG, Immunoglobulin G; OR, Odds Ratio; SARS-CoV-2, Severe Acute Respiratory – Coronavirus – 2.

^a^Medium and high tertiles are compared with the lowest tertile of IgG level in an unconditional logistic regression model.

^b^P value from a likelihood ratio test for trend.

**Sensitivity Analysis 5:** All Umoya cases (n=14) and asymptomatic controls (n=9)

| Table. Unadjusted estimates of the association between SARS-CoV-2 IgG serostatus and pulmonary TB disease for the Umoya study. | | | | | | |
| --- | --- | --- | --- | --- | --- | --- |
| Dataset | **Participant group** | **SARS-CoV-2 IgG seropositive/ seronegative** | **Unadjusted OR**  **(95% CI)** | **p-value** | **Adjusted OR^a^**  **(95% CI)** | **p-value** |
|  |  |  |  |  |  |  |
| Umoya | Non-TB controls | 3/6 | 1.0 |  | - |  |
|  | TB cases | 4/10 | 0.80 (0.13 – 4.87) | 0.81 | - | - |
|  |  |  |  |  |  |  |

Abbreviations: CI, Confidence Interval; IgG, Immunoglobulin G; OR, Odds Ratio; SARS-CoV-2, Severe Acute Respiratory Syndrome-Coronavirus-2; TB, Tuberculosis.

^a^Adjusted OR not calculated due to too few outcome events per predictor variable.

All p-values calculated from a likelihood ratio test

| Table. Odds of pulmonary TB by SARS-CoV-2 immunoglobulin G levels^a^ | | | |
| --- | --- | --- | --- |
| Viral-specific IgG level | **Sample, No.** | **Unadjusted OR (95% CI)** | **p-value for trend^b^** |
| SARS-CoV-2 |  |  |  |
| Low (154.20 – 249.99 AU/ml) | 2 | 1.0 (Reference) | 0.18 |
| Medium (209.50 – 488.69 AU/ml) | 2 | 1.00 (Empty) |  |
| High (488.70 – 2598.40 AU/ml) | 3 | 1.00 (Omitted) |  |
|  |  |  |  |

Abbreviations: AU, Arbitrary Units; CI, Confidence Interval; IgG, Immunoglobulin G; OR, Odds Ratio; SARS-CoV-2, Severe Acute Respiratory – Coronavirus – 2.

^a^Medium and high tertiles are compared with the lowest tertile of IgG level in an unconditional logistic regression model.

^b^P value from a likelihood ratio test for trend
